# A Golden Thread approach to transforming Maternal and Child Health in Singapore

**DOI:** 10.1186/s12884-022-04893-8

**Published:** 2022-07-14

**Authors:** Fabian Yap, See Ling Loy, Chee Wai Ku, Mei Chien Chua, Keith M. Godfrey, Jerry Kok Yen Chan

**Affiliations:** 1 Department of Pediatrics, KK Women’s and Children’s Hospital, Singapore, 229899 Singapore; 2 Duke-NUS Medical School, Singapore, 169857 Singapore; 3 Lee Kong Chian School of Medicine, Nanyang Technological University, Singapore, 636921 Singapore; 4 Department of Reproductive Medicine, KK Women’s and Children’s Hospital, Singapore, 229899 Singapore; 5 Department of Neonatology, KK Women’s and Children’s Hospital, Singapore, 229899 Singapore; 6 Medical Research Council Lifecourse Epidemiology Unit, University of Southampton, Southampton, SO16 6YD UK; 7 National Institute for Health Research Southampton Biomedical Research Centre, University of Southampton and University Hospital Southampton National Health Service Foundation Trust, Southampton, SO16 6YD UK; 8 Yong Loo Lin School of Medicine, National University of Singapore, Singapore, 119228 Singapore

**Keywords:** Healthcare framework, Life-course, Maternal and child Health, Mental health, Metabolic disease, Non-communicable disease, Singapore, Transformation

## Abstract

Maternal and child health (MCH) in Singapore is entering a new phase, with challenges different to those faced 50 years ago. The advancement of medical technologies and access to MCH resources have led to a dramatic fall in maternal and infant mortality rates. However, there has been a steep rise in the rates of obesity and related metabolic diseases. Alongside this is an emerging mental wellness challenge, with one in ten women experience depression across pre-, during and post-pregnancy. Maternal obesity and mental disorders before and during pregnancy not only increase a woman’s risk of pregnancy complications, but also result in increased risks in the offspring of childhood obesity, behavioral disorders and later life metabolic disease, catalyzing vicious cycles of disease. Thus, there is a pressing need to transform the current MCH system to address a burgeoning metabolic and mental health challenge for Singapore. Initiating interventions during preconception and continuing into the postpartum has the potential to confer long-term maternal-child benefits, promoting virtuous cycles of health. However, the current MCH system emphasizes antenatal care and lacks focus on the equally, if not more important, preconception, postpartum and inter-pregnancy stages. We describe a new model-of-care framework that integrates a life-course approach to health across preconception, pregnancy and postpartum phases, with the social-ecological model comprising individual, interpersonal, institutional, community and policy as the major targets for health promotion interventions. This “golden thread” approach is being established at the Singapore KK Women’s and Children’s Hospital (KKH), to address both metabolic and mental health challenges to achieve the goal of a thriving, healthy nation. This new model-of-care is set up in KKH as a pilot program known as Healthy Early Life Moments in Singapore (HELMS). HELMS will reach out to women planning to conceive through coordinated interventions across preconception, pregnancy and postpartum periods. A mobile health platform is being developed to facilitate interventions and engage participants in the program through a digital, personalized and interactive approach. This new model-of-care is designed to secure a population with healthy life cycles, by influencing each life-course, early-in-life, to provide the best start for generations to come.

## Background

Maternal and child health (MCH) forms the bedrock of healthy families and societies [1]. Improving MCH is vital for all countries, but particularly for Singapore, because of rapid socio-economic development, ageing and ultra-low fertility rates [2, 3]. Over the past 50 years, Singapore has made remarkable achievements in MCH, substantially lowering maternal and infant mortality rates. Maternal mortality in Singapore fell from 49.4 to 8.0 cases per 100,000 live births from 1966 to 2017 [4, 5], while infant mortality rate fell from 27.4 to 2.1 cases per 1000 live births from 1965 to 2019 [6]. The Singapore government’s approach to MCH transformation as an integral part of the overall development planning for the country has been key to the progress in this area.

**Fig. 1:**
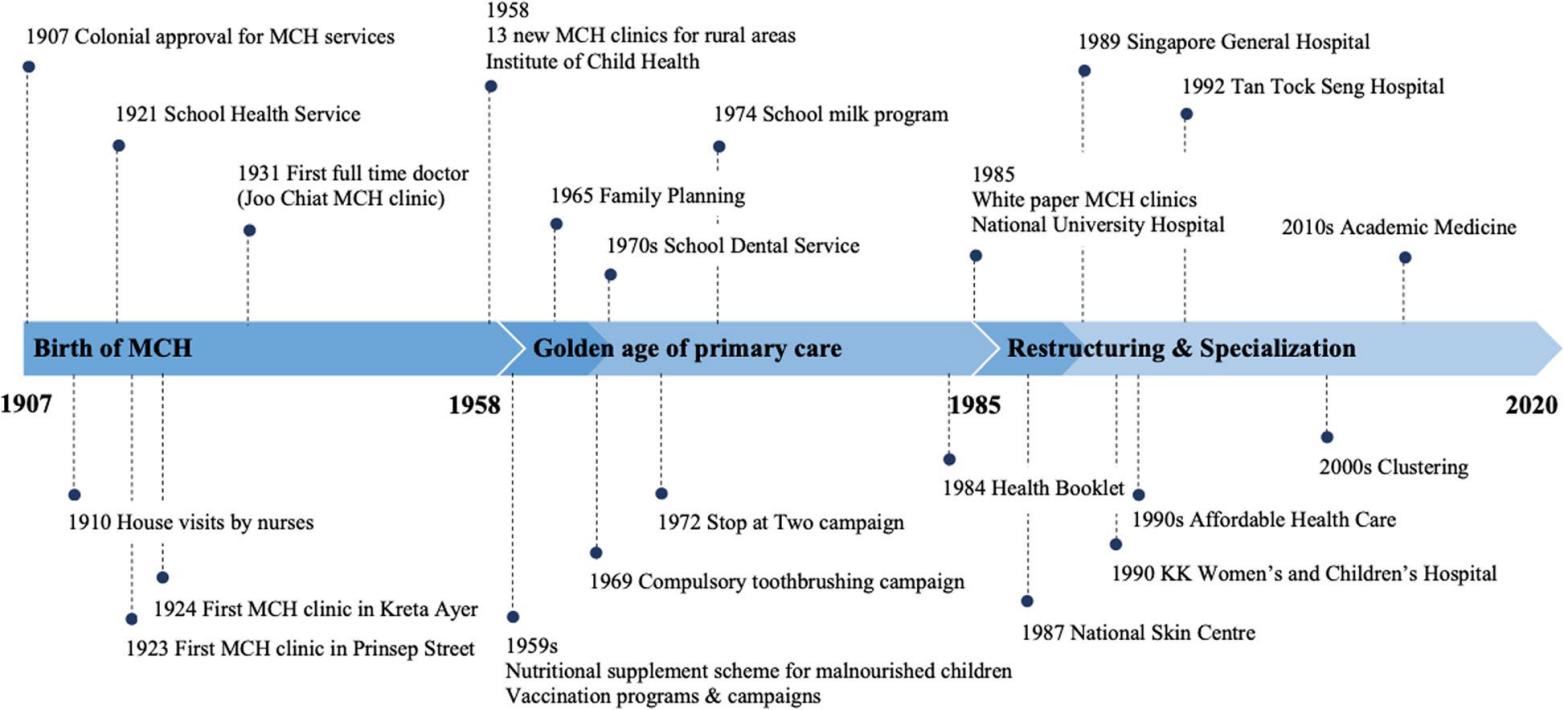
Evolution of the maternal and child health system in Singapore**.** The development of the MCH system in Singapore between 1907 and 1957, 1958–1984 and 1985–2020. MCH, maternal and child health

The development of the MCH system in Singapore can be divided into three time periods – 1907-1957, 1958–1984 and 1985–2020 (Fig. 1). At the turn of the twentieth century, primary MCH care was established to address undernutrition and infectious diseases that caused women and children to die [7]. This began with the set-up of treatment centers from which midwives could be dispatched to deliver babies and provide advice on childcare, especially for those in the rural areas. This effort was transformed with nurses performing house-to-house visits [8]. Simultaneously, MCH clinics started to be established in the urban areas and slowly expanded across the country [8]. Fuelled by a demand for midwifery and infant care services, progressive development of nurse-driven, clinic-based MCH services extended into the mid-twentieth century [8]. Current primary MCH services are now delivered in 20 polyclinics and 1700 general practice clinics island wide [9]. When survival rates improved, fertility rates rose and the medical needs for women and children became more complex. Consequently, the demand for hospital-based care grew stronger, leading to the establishment of departments specializing in obstetrics and paediatrics that drove physician-led MCH care within general hospitals [8]. Later, in the second half of the twentieth century, further care demands of women and children gave rise to the sub-specialties of maternal-fetal medicine and neonatology, respectively [8]. During this period, as institutions became preoccupied with supporting the insatiable demand for higher quality systems, medical attention gravitated towards assisted reproductive techniques, alongside management of complicated high-risk pregnancies and complex childhood diseases.

Today, Singapore faces new MCH challenges as the nation’s attention shifts from “survive to thrive”, and from “healthcare to health” [10]. Achievements of the past, in terms of reduced maternal and infant mortality rates, have now been overshadowed by the sharp increase in metabolic disorders and non-communicable diseases (NCDs) [11], mainly arising from high rates of obesity [12]. The prevalence of obesity [body mass index ≥30 kg/m^2^] among Singaporean women is increasing, from 6.1% in 1992 to 10.3% in 2017 [13] and is projected to reach 15.9% in 2050 [14]. This has far-reaching implications for women of reproductive age. Obesity before and during pregnancy not only increases the risk of subfertility and adverse pregnancy outcomes, but also puts the child at risk of developing childhood obesity and related NCDs in later adult life, establishing a vicious cycle of obesity-related diseases [15, 16]. Likewise, maternal mental health disorders, including anxiety and depression, are also on the rise, with adverse impacts on long-term child behavioral development and cognitive function [17]. Up to one in 10 women experience depression during preconception, which continues into the pregnancy and postpartum periods [18, 19]. Emerging evidence shows that the children of mothers with depressive symptoms during pregnancy are at risk of altered structural brain development, as well as poor school-readiness and performance in early childhood [19, 20].

**Fig. 2:**
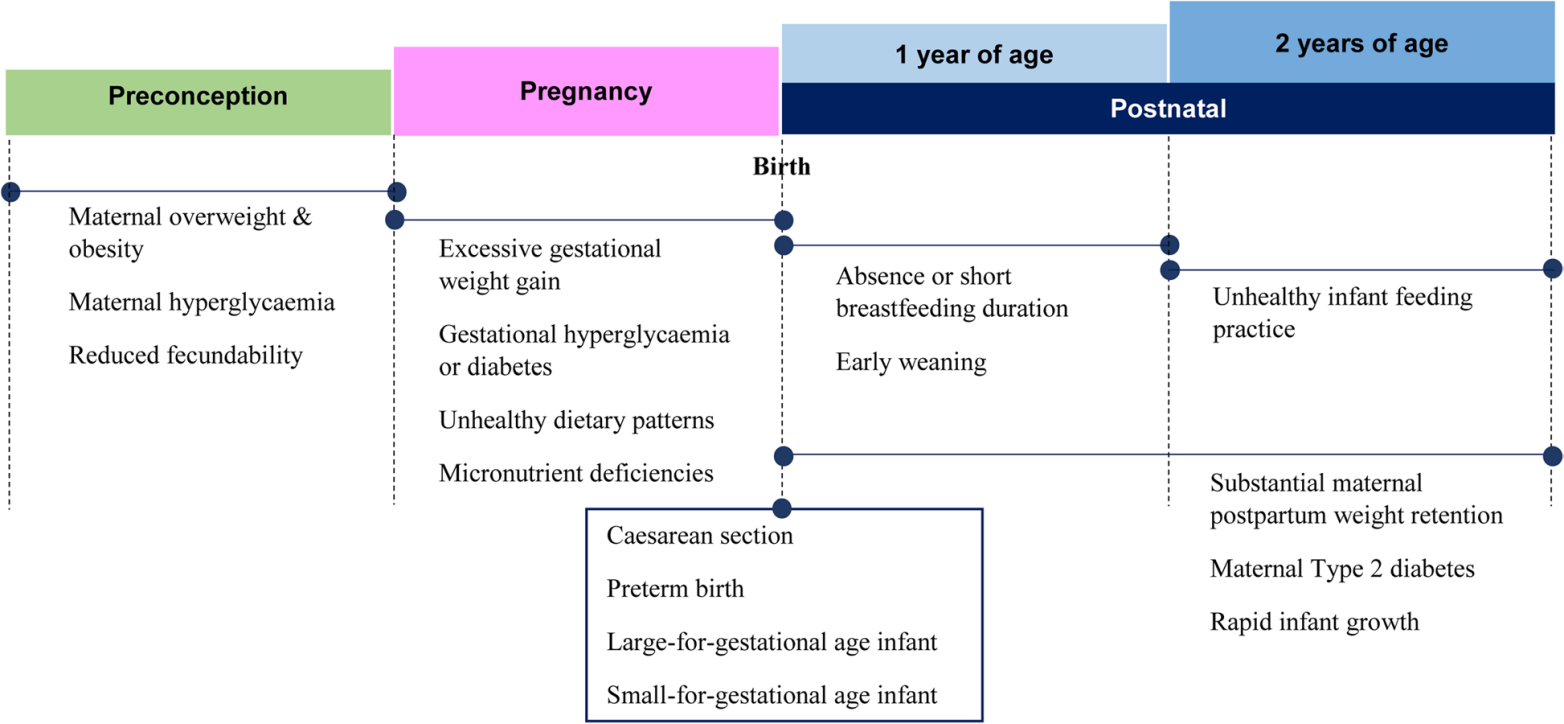
Maternal obesity and its complications across different life stages**.** Findings from the S-PRESTO and GUSTO studies. At preconception, women who were overweight or obese were at-risk of hyperglycemia and reduced fecundability. When entering pregnancy, these women were more likely to experience excessive gestational weight gain and develop gestational diabetes, which were associated with adverse delivery and birth outcomes. Children born to obese mothers were susceptible to low breastfeeding exposure, early weaning and accelerated weight gain, placing them at risk of early childhood obesity and metabolic disorders. Meanwhile, women themselves had increased risks of substantial postpartum weight retention and type 2 diabetes development. S-PRESTO; Singapore PREconception Study of long-Term maternal and child Outcomes; GUSTO, Growing Up in Singapore Towards healthy Outcomes

Emergence of these twin “metabolic and mental health” challenges in Singapore has been made evident from two mother-child prospective cohort studies, namely the Singapore PREconception Study of long-Term maternal and child Outcomes (S-PRESTO) and the Growing Up in Singapore Towards healthy Outcomes (GUSTO) studies [21, 22]. We illustrate in Fig. 2 an overview of maternal obesity and its complications for mother and child across different life stages, based on findings from these cohort studies [23–32]. The findings highlight the pressing need to arrest the trajectory of increasing health risks across life stages and generations. Since behavioral interventions commenced during pregnancy have limited positive impact on maternal fetal outcomes such as gestational diabetes or macrosomia [33, 34], optimizing preconception health to promote optimal embryo and fetal development is now widely recognized as the key to improving health outcomes for both mother and child [35]. Starting interventions preconception, in particular for women who are obese, has the potential to confer long-term maternal-child benefits and promote virtuous cycles of health [36]. The current MCH system places principle emphasis on antenatal care and lacks focus on the equally important preconception and postpartum care. Therefore, this paper aims to discuss: 1) the current clinical challenges and needs of the Singapore MCH system, and 2) the creation of the framework for a new model-of-care to address the twin “metabolic and mental health” challenges to achieve the goal of a thriving, healthy nation.

### Gaps and needs in the current MCH system in Singapore

Traditionally, Singapore’s MCH system overly emphasizes disease rather than health, resulting in a lack of knowledge and attention given to preventive medicine. This is especially relevant to metabolic and mental health, throughout preconception, pregnancy, and postpartum life stages. As a result, women are generally ill-prepared for pregnancy, which is compounded by a lack of preconception health services in the current MCH system. In many developed countries, including Singapore, women’s health services are structured around prevention of unintended pregnancy, management of infertility, or screening and managing medical disorders in pregnancy through routine antenatal visits [1]. While these are important medical services, the clinical need to improve preconception metabolic and mental health is unmet, leaving the outcome of a successful and healthy pregnancy to nature and to chance.

The lack of preparedness carries on after the baby is born. During the immediate postpartum period, a woman’s own needs are often neglected when the attention shifts to caring for the newborn. This new phase in life brings about unexpected physical and emotional challenges that may culminate in perinatal mental disorders. One of the most common morbidities during pregnancy and the perinatal period, mental disorders contribute significantly to maternal mortality and adverse child outcomes [37]. When not properly treated and managed, women may enter the next pregnancy in poorer metabolic and mental health, perpetuating vicious health cycles. For the child, it is often assumed that parents and caregivers have the knowledge and skill to provide optimal nutrition and to inculcate healthy eating behavior. The reality could not be further from the truth. For example, consumption of cakes, biscuits and snacks were found to contribute to a high percentage of energy to infants, especially in those who were breastfed [38]. This highlights the momentous clinical challenge involves the establishment of healthy feeding and nutrition milestones. Current infant health services are largely focused on the provision of vaccinations, neurodevelopment screening and management of acute illnesses, with little emphasis on age-appropriate weaning practices and supporting healthy development. Singapore needs a system of care to provide optimal nutrition guidance to establish healthy eating habits throughout infancy and childhood.

**Fig. 3:**
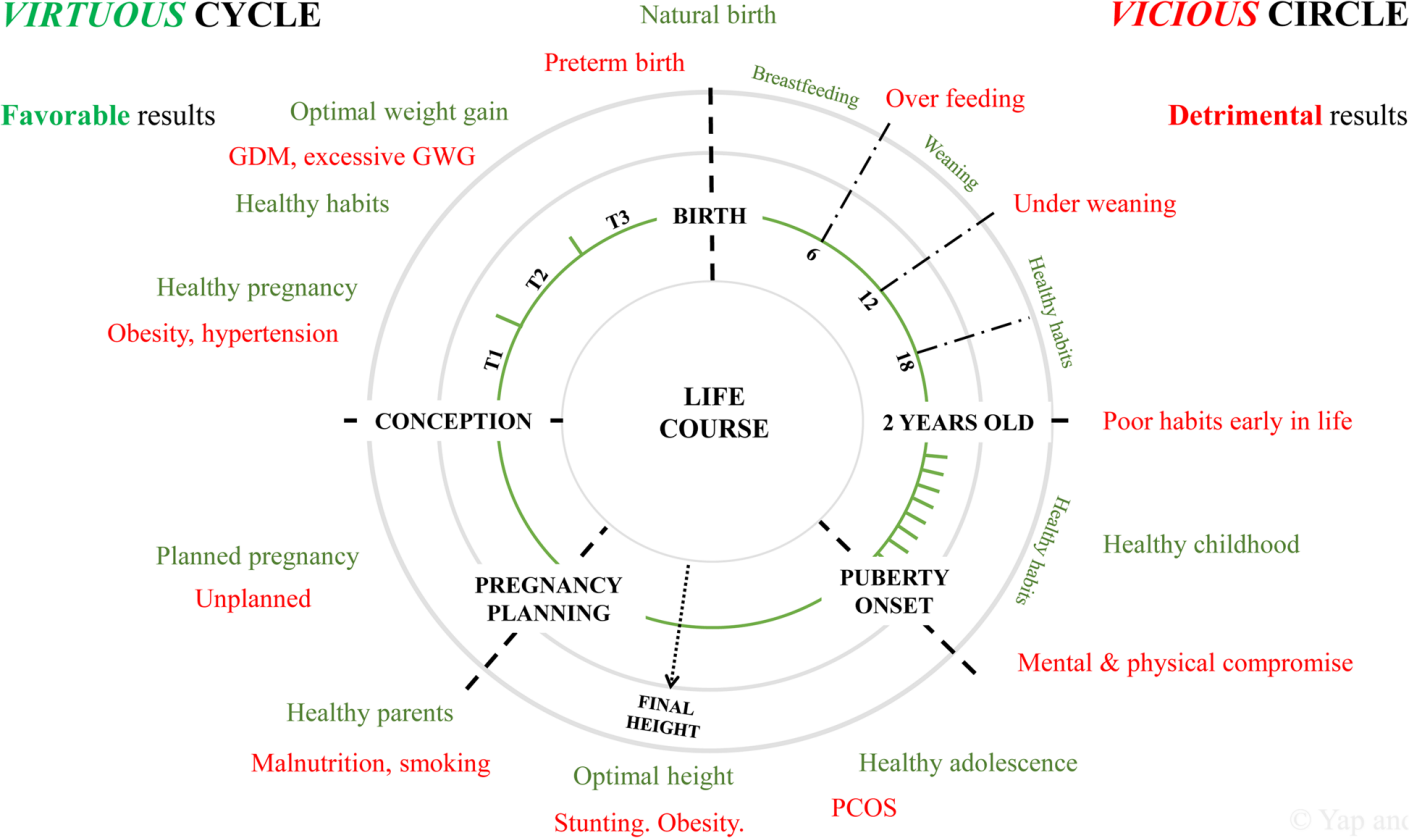
Vicious and virtuous cycles of health**.** Vicious (in red) and virtuous forces (in green) of obesity and non-communicable diseases development. GDM, gestational diabetes mellitus; GWG, gestational weight gain; PCOS, polycystic ovarian syndrome; T1, first trimester; T2, second trimester; T3, third trimester

To address these challenges in the current MCH system, the pyramid of care in maternity will need to be inverted to tackle potential problems further upstream, starting from preconception. This will serve to optimize health and improve reproductive potential [36], while preventing social and health implications due to unplanned pregnancies. Preconception lifestyle interventions have demonstrated positive maternal behavioral changes such as supplementation compliance, healthy dietary intake, increased physical activity and smoking cessation [39, 40]. Women who successfully lose weight during such interventions themselves had better cardiometabolic health **6** years later [40]. Such promising results pave the way for a paradigm shift where public health efforts target women who are planning for pregnancy, to provide the best environment for fetal development. Figure 3 represents a possible roadmap for transitioning from a vicious to a virtuous cycle of health.

**Fig. 4:**
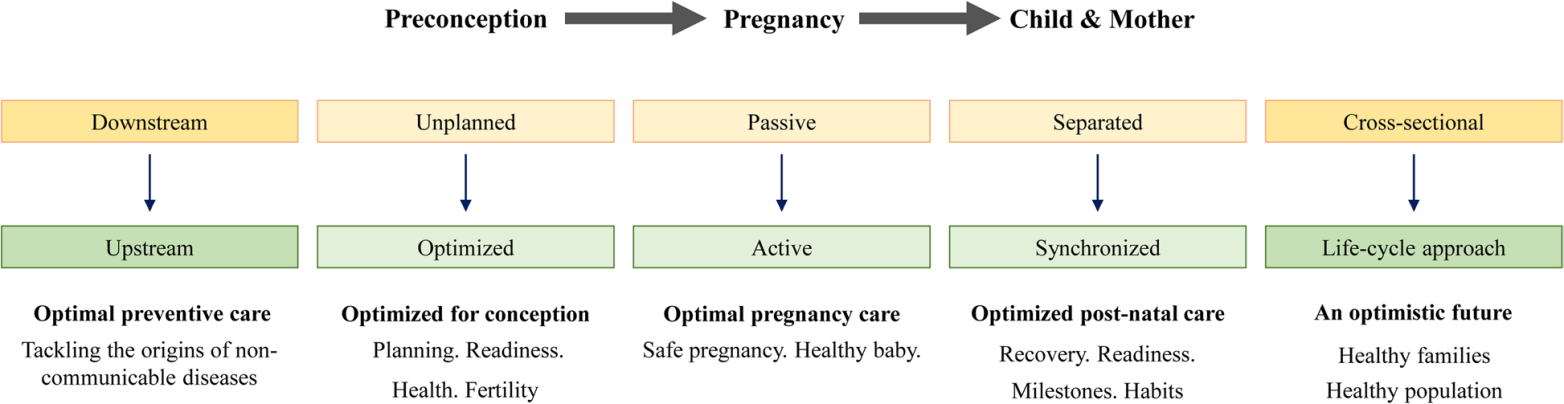
Optimizing potential through a continuum of care model. Continuum of care acting upstream from preconception, through pregnancy until postpartum period involving mother-child dyad pair

The empowerment and education of women, their spouses, and parents in preparation for pregnancy and childbirth are crucial for the well-being of both mother and baby, which involve modifying current antenatal care services. Changes to postpartum maternal care must focus on optimizing recovery from childbirth and ensuring readiness for the next pregnancy, while infant care must involve purposeful feeding guided by growth monitoring. The importance of this phase has now been coined the fourth trimester for the child and matrescence for the woman [41]. These strategies form a basis to promote virtuous life cycles of health. Singapore’s past initiatives that aimed at improving maternal and child health mainly focused on specific life stages and were not designed from a life cycle perspective to address root causes of metabolic and mental disorders. Since 2017, the healthcare system in Singapore has focused on ‘The 3 Beyonds’ – (i) beyond healthcare *to health*; (ii) beyond hospital *to community*; and (iii) beyond quality *to value* [10]. We envision an integrated life-course approach in a care continuum that encompasses preconception optimization, pregnancy participation, and postpartum synchronization of maternal-child health services in the first **2** years of life (Fig. 4). Threading together the preconception, pregnancy and postpartum journeys will achieve synergy in producing greater behavioral change and beneficial outcomes not only for the woman, but also for her child and family. This movement aligns with the national effort on population health, in particular, enhancing upstream preventive efforts to promote health for women and children. In 2021, the authorities set up a task force on Child and Maternal Health and Well-being, and subsequently announced a new preventive care strategy for Singapore or Healthier SG in 2022, as major platforms to address issues associated with MCH via multi-agency collaborations [42–44].

### Repurposing the reproductive, maternal, newborn and child health framework from “survive to thrive”

Reproductive, maternal, newborn, and child health (RMNCH), which encompasses health concerns spanning the life-course of women before, during and after pregnancy, and their newborns and children, is a key priority for lower- and middle-income countries where the main goal is to prevent deaths and stillbirths [1]. In higher income countries such as Singapore, optimizing health and human potential is now a high priority. We seek to develop a new RMNCH model-of-care that integrates the contemporary health needs of women, with the health benefits for their children throughout the life-course. A multidisciplinary team comprises healthcare professionals from obstetrics and gynecology, neonatology and pediatrics, dietitians, psychotherapists, and nurses will be involved to deliver this integrated care. We envision that a holistic approach to health, embracing the pillars of metabolic and mental health that is primarily built on preventive care, will promote a culture of general well-being, productivity, and equity. This immense task requires the creation of new healthcare models that are modular and deployable across health systems, aligned with Singapore’s 2017 healthcare system transformation effort known as ‘The 3 Beyonds’, as aforementioned.

**Fig. 5:**
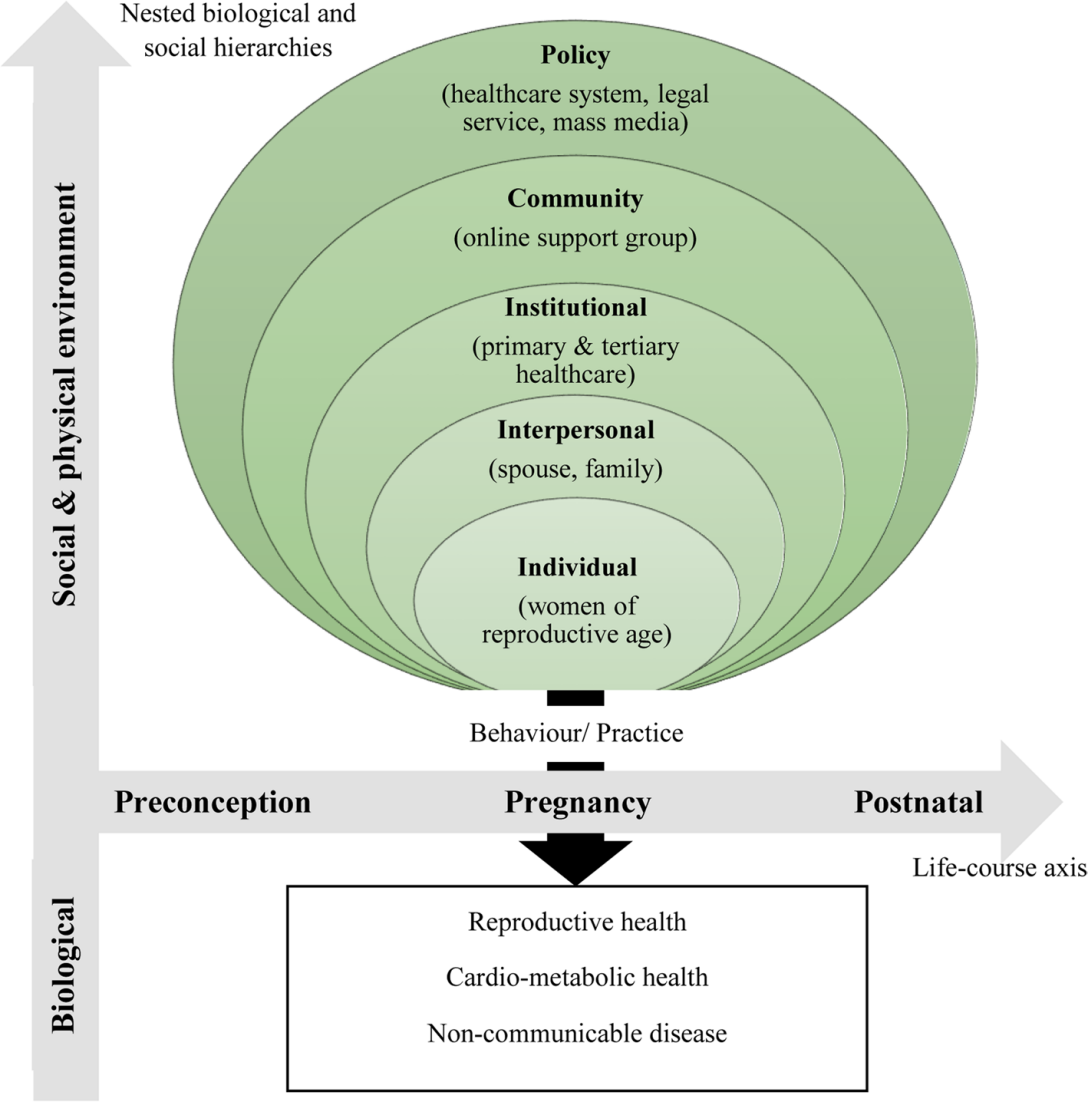
Interaction between environment and behavior. This model illustrates the design of interventions at multiple levels (social and physical environment) that can influence women’s behaviors and practices, and thus health (biological environment) across preconception, pregnancy and postnatal phases. Modified from Glass et al. [46]

To determine the major targets for health promotion interventions, we adopted a social-ecological model for health promotion that comprises a holistic multi-level framework involving – the individual, interpersonal, institutional, community and policy [36, 45]. Women play multiple roles in modern society – as individuals, mothers, wives and working professionals. Their interactions with others at interpersonal, organizational and community levels may culminate in numerous factors that support either healthy or unhealthy behaviors. For the latter, appropriate changes in the social environment using a bottoms-up approach, coupled with public policies that support healthy behaviors from a top-down approach, represent the most viable and sustainable way to implementing this new model-of-care. Figure 5 illustrates a model, modified from Glass et al. [46], to show the interaction between environment and health behavior.

### The individual as the key stakeholder

The woman is at the heart of the new RMNCH model-of-care. As we strive to provide the most patient-centric care, there is also a need to guide women through the hazards of risk factors across the life-course. Interventions including nutrition, physical activity and mental wellness should be sustained throughout the key stages from preconception to postpartum life. The reproductive years of a woman’s life-course represent a good opportunity for the promotion of their health and that of their offspring. Continuous lifestyle support before and during pregnancy can empower women and mothers-to-be with knowledge to make informed decisions on how to give their baby the best start in life. Extension of lifestyle support into the postpartum phase helps women maintain a healthy mindset, facilitate behavioral change that can influence child feeding practices, and improve the recovery process from childbirth, specifically physical (e.g. weight) and physiological recoveries (e.g. glucose metabolism).

### Interpersonal relationships

The extent to which individuals can sustain their behavioral changes is largely influenced by their social environment, in particular, support from close relationships. For example, the likelihood of a woman engaging in physical activity or adhering to supplementation during pregnancy was shown to be strongly influenced by having a spouse who was physically active and family members who reminded and encouraged them [47, 48]. Building strong relationships before, during and after pregnancy not only serves to motivate women in their healthy lifestyle journey, but also promotes greater mental resilience and mitigates the risks of mental health problems during these stressful changes in family life.

### Institutional factors

Collaboration and partnerships between primary care and specialist services are key to tackling obesity, diabetes and other NCDs. This paradigm shift needs to be supported with education and training for healthcare workers in primary and tertiary care, as both have important roles to play in ensuring sustainability and seamless transition from preconception to pregnancy and postpartum dyad care in this new model-of-care. Inclusion of academic curricula that explain the underlying concepts leading to the need for the new approach has the potential to increase acceptance of this new model among staff and facilitate leadership development during implementation. Equipping health and social care staff, especially those from primary care settings, with knowledge and skills to support lifestyle behavioral change in the community, is especially pertinent during the preconception and postpartum phases.

### Community engagement

Technological advances play a key role in transforming service delivery in the community and on a nationwide level. Online community social platforms targeting a particular group have emerged as an important channel for the delivery of lifestyle and mental health support along the continuum of care [49]. For example, it is common for pregnant mothers to come together to share their experiences, identify common problems in child preparation or childrearing, and generate solutions that work for them in online chat groups [50]. Digital engagement via story sharing has shown to be effective for health promotion in communities [51].

### Policy support

Generating political will for healthcare policy and RMNCH system change necessitates substantial advocacy activity to reach the majority of the population, which again needs to be driven by public education, awareness and demand. To this end, the Singapore Ministry of Health recently announced the development of a Child and Maternal Health and Well-being Strategy to provide comprehensive support to women and their children [42, 43]. The Strategy and Action Plan will be overseen by a multi-agency task force including the Ministry of Health, Ministry of Education and Ministry of Social and Family Development, over a five-year period. The scope will span from preconception to adolescents aged 18 years. This Action Plan was developed in recognition of the need to enhance upstream preventive health efforts for women and children, with a greater integration of social and health services across agencies, to ensure timely delivery of health services to both mother and child. This is in conjunction with the broader scope of adopting a life-course approach to population health in Singapore, with the Healthier SG strategy, which includes mobilization of family physicians and community partnerships to support better health, with support structures and policies to be put in place over the next few years [44]. Besides integrating services for the mother and child, the integration of services across the health and social domains for more holistic support for children and their families ensures better access to health for everyone.

### The Healthy Early Life Moments in Singapore (HELMS) program

The KK Women’s and Children’s Hospital (KKH) is an academic medical center in one of the three main Health Care Clusters in Singapore, responsible for one-third of the nation’s birth. With that, KKH is ideally positioned to lead the development and implementation of an integrated model-of-care to improve the health of both women/mothers and children. We leverage on our clinical specialties and large population of approximately 12,000 births annually, to design and detail a new RMNCH framework of care, which we will deliver as the HELMS pilot program. HELMS represents the clinical translation and implementation of lessons learnt from the S-PRESTO and GUSTO cohort studies [21, 22]. Its main goal is to improve the health and outcomes of overweight and obese women who are planning for pregnancy, as they constitute the group at greatest risk of metabolic and mental health problems. The longer-term goal is to provide HELMS care to all women before, during and after pregnancies (along with their children). This is in line with the strategic thrust for maternal and child health outlined in preceding paragraphs.

HELMS was designed with patients in mind, recognizing their pivotal role in a successful lifestyle intervention program. In September 2020, the HELMS team initiated in-depth interviews with women who were in the preconception, pregnancy or postpartum stages, to understand their needs and personal factors that would influence their participation and engagement in an intervention program [52]. In our dialogue with participants, we recognized that women had poor knowledge (on child health consequences of maternal obesity) and were not aware of reliable sources for health information. They indicated a strong desire to receive continuous guidance from healthcare professionals while planning, undergoing and recovering from pregnancy. They wanted mobile health (mHealth) platforms as a means of intervention. The information gathered from these interviews was crucial in informing and guiding the development and implementation of HELMS.

**Fig. 6:**
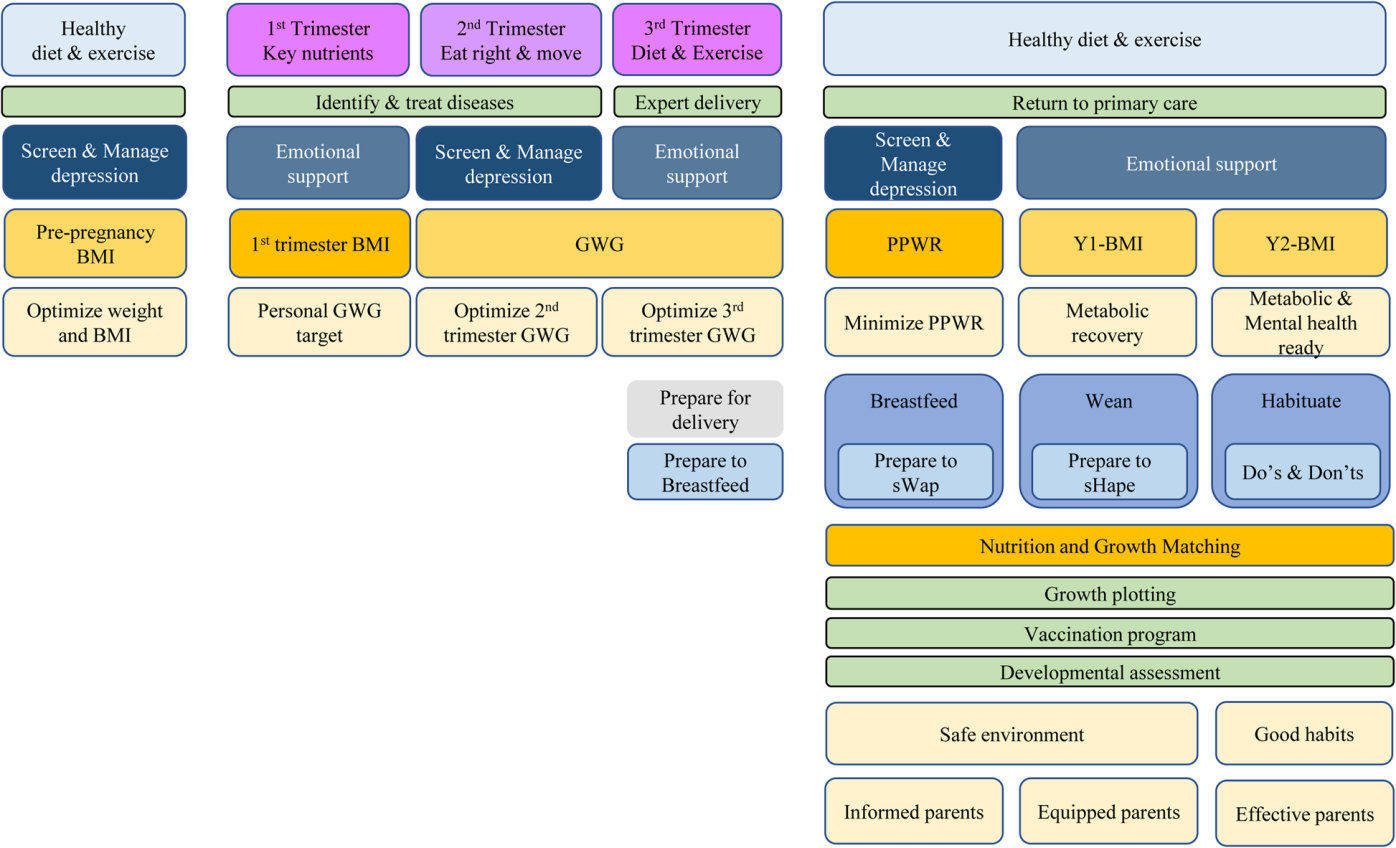
Healthy Early Life Moments in Singapore model-of-care**.** A new model-of-care framework for Reproductive, Maternal, Newborn and Child Health based on a life-course approach and delivered on a mobile health platform. BMI, body mass index; GWG, gestational weight gain; PPWR, postpartum weight retention. Boxes in green represent the existing care model; boxes in other colors represent the enhanced care model

HELMS aims to address the current two major challenges to RMNCH in Singapore, i.e. metabolic and mental health disorders, through a suite of interventions, with the ultimate aim of providing the best and most equal start to life for our children. We adopt a life-course approach in the program, which recognizes the additive effects of influences in a person’s life that will impact the eventual health trajectory. HELMS is based on the traditional twin principles of justice and égalité, and addresses healthcare equity by ensuring a positive and equal start to life for our children via upstream preventive care enhancement for women and children, and better delivery of RMNCH service to enhance convenience for families. It aims to impart new mental models of nutrition, physicality, and mental wellness, by guiding, supporting and empowering women through an integrated journey from preconception to postpartum life stages, to address metabolic and mental health challenges. Women at high risk of mental health issues, or other medical conditions and comorbidities such as preexisting diabetes or hypertension, will be referred to the appropriate specialists for ongoing management. HELMS forms the foundation of transforming current healthcare structure by integrating a clinical service line for RMNCH. Figure 6 illustrates an overview of the HELMS program, focusing on NCD prevention and mental health promotion through a range of interventions. The delivery of care and intervention at each phase will be performed by maternal and child healthcare professionals including clinicians, dietitians, physiotherapist, nurses and trained clinical staff, with the aid of mHealth technology. The use of mHealth platform is demanded by the target groups and supported by the high smartphone penetration in Singapore with up to 96% smartphone access rate among our population in 2021 [53, 54].

### HELMS model-of-care

In the pilot phase (clinicaltrials.gov, NCT05207059), HELMS will engage overweight and obese women from preconception for one year, followed by pregnancy until 18 months postpartum, via study visits that align with their routine clinical follow-ups, and through a mobile health (mHealth) platform. We use a **4S** strategy to implement care: ‘**S**creening’, ‘**S**ize’, ‘**S**upplementation’ and ‘**S**pecial considerations’. **Screening** involves health and risk assessments through physical examination and biomarker measurements (e.g. anti-mullerian hormone, glucose, insulin and lipid profile), as well as eating habits, exercise, emotion and sleep evaluations. Triaged care for high-risk women is provided based on the real-time performance feedback from the digital screening tool. Body **size** management encompasses healthy eating education, monitoring physical activity and tracking personal weight. **Supplementation** includes multi-micronutrients, vitamin D, calcium and/or DHA supplements, which varies across phases and targets (mother and child). **Special** considerations comprise management of preconception sexual health and function, pregnancy symptoms, birth recovery, infant growth monitoring and feeding. The establishment of mother and baby friendly initiatives sets the stage for optimal dyad care right from the start. This includes one-to-one midwifery care during labor, skin-to-skin contact at delivery, breastfeeding initiation and support in accordance to the ten steps to successful breastfeeding for baby-friendly hospitals.

mHealth education in English, which is the main language in Singapore, will be developed to focus guidance on healthy eating, exercise, sleep and mental well-being, in addition to increasing knowledge regarding preconception, antenatal and postpartum dyad care. A digitized tool that aims to address metabolic heath issue based on the principle of energy input and expenditure, and motivation as the foundation to make dietary change is designed [55]. It comprises dietary, activity and psychological components, which serves as a check list to facilitate an individual to be aware of own unhealthy lifestyle problems and tackle it via SMART (Specific, Measurable, Achievable, Realistic and Timely) strategy. For mental health and sleep, collective tips on relieving stress, building mental resilience and practicing good sleep hygiene are provided and accessible online. In general, HELMS is aligned with Singapore’s dual move to digitize the nation, and optimize human health and potential (National Research Foundation’s Research Innovation Enterprise 2025’s mission).

**Fig. 7:**
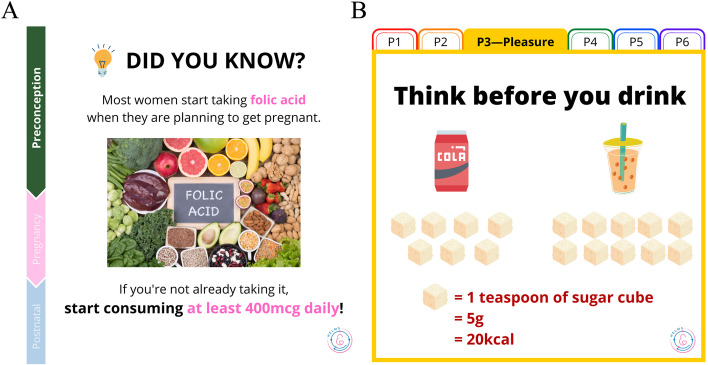
Mobile health messages. Samples of mobile health messages that serve as nudges which are phase-specific (**A**) and diet-specific (**B**)

HELMS will apply a **SIGN** approach (**S**upport, **I**nform, **G**uide and **N**udge) to deliver the clinical care, healthy lifestyle and behavioral supports via mHealth. Close monitoring of women’s health allows timely **support** to be provided by healthcare staff. To effectively support women to make lasting lifestyle behavior changes, especially in the area of nutrition and physical activity, HELMS staff will adopt Healthy Conversation Skills. This technique has been proven successful at increasing the confidence and competence of front-line staff to have productive conversations with patients on making positive behavioral changes [56]. During pregnancy, by leveraging on the present antenatal service, every antenatal visit presents a unique opportunity to educate the woman and her family on healthy lifestyle. To build community support, digital social support groups for story sharing will be formed based on topics of interest, such as healthy home cooking, antenatal exercise, breastfeeding support and child growth. The setup of mHealth education is a convenient way to **inform** women of the importance and points of caution at different phases. Educational materials in various formats, including diagrams and videos will be developed to provide **guidance** on phase-specific care and healthy lifestyles focusing on nutrition, physical activity, mental health and sleep hygiene. **Nudges** in the format of image-enhanced text messages that are tailored to women’s life-course stages and self-selected lifestyle goals (e.g. eating habit and exercise) will be designed to educate, remind and motivate women to sustain behavioral changes. Samples of developed nudges are illustrated in Fig. 7. The use of nudges represents a preferred architecture strategy which is widely used in public policy making, to support positive behavior changes and influence decision making [57].

The mHealth intervention components in HELMS include educational materials delivered in a phase-specific fashion across the life-course, community social platform, lifestyle assessment surveys with real-time performance evaluations and recommendations, goal settings for making behavioral changes based on the evaluations, phase- and goal-specific nudges delivery, tracking calendars for weight and supplementation, and monitoring charts for lifestyle activities including dietary intake, intensity of exercise, presence of depressive symptoms and sleep performance. Taken together, we employ a personalized, interactive multi-modality approach to meet individual needs, and enhance participation interest, engagement and compliance of women throughout the HELMS program. To evaluate the success of the program, various clinical (e.g. metabolic and mental health, fertility, pregnancy complications and child growth) and implementation outcomes (e.g. cost-effectiveness and acceptability) will be measured and compared with an independent observational preconception cohort in Singapore with similar timepoints and outcome measures [21].

## Conclusion

We present a “golden thread” journey from strategy to delivery from the Singapore perspective. The successful transformation of our current MCH system into a life-course integrated model-of-care, starting with the HELMS pilot program in a single institution, requires strong collaboration at the individual, interpersonal, community, institutional and national levels to generate awareness and promote cooperation. Providing a continuum of care across the early life-course, starting from preconception, pregnancy until the postpartum period, builds a strong foundation for healthy women and secures a healthy start for future generations. This would pave the way towards achieving the global Sustainable Development Goals, in terms of health, well-being, economic, humanitarian and equity benefits, addressing the nation’s goal beyond healthcare to health as Singapore transitions from a mindset of survival to an emphasis on thriving.

Although HELMS will begin by engaging preconception women who are overweight or obese, we envision that the entry point of the program can be at any phase and includes women who are not overweight, those with metabolic disorders or with polycystic ovary syndrome. This modular approach allows flexibility and scalability of the program, which is crucial for the transferability and application of the HELMS intervention to a nationwide primary care setting (e.g. subsidized polyclinics) after the pilot phase. Under this initiative, women and children will be systematically screened for both health and social needs when visiting polyclinics for HELMS service care. Reallocation of healthcare resources, investment in staff training, establishment of community care plans and services, as well as epidemiological and implementation science research are essential components to scale up the HELMS and fully transform the current MCH system. Partnership with voluntary welfare or philanthropic organizations will be progressively built to extend efforts and reach in the community, including vulnerable and ethnic minority groups through enhancement of HELMS program delivery and social support. Further, technical advancement of HELMS digital platform is required by having multilingual support function and with modules that are compatible across common digital tools to ensure program accessibility to the nation. Collaboration with Infocomm Media Development Authority, which seeks to drive Singapore’s digital transformation, is essential to equip low-income patients with digital tools for healthcare purposes. To be successful and sustainable, we will need multi-sectorial support and skillful change management to overcome anticipated roadblocks and obstacles. Most of all, we need to be inspired by a collective purpose – to secure a population with healthy life cycles, by influencing each life-course, early in life.

## Data Availability

The original contributions presented in the study are included in the article, further inquiries can be directed to the corresponding author.

## Abbreviations

MCH: Maternal and child health
KKH KK: Women’s and Children’s Hospital
HELMS: Healthy Early Life Moments in Singapore
NCD: Non-communicable diseases
S-PRESTO: Singapore PREconception Study of long-Term maternal and child Outcomes
GUSTO: Growing Up in Singapore Towards healthy Outcomes
RMNCH: Reproductive, maternal, newborn, and child health
mHEALTH: mobile health
